# Less might be more: 1 mA but not 1.5 mA of tDCS improves tactile orientation discrimination

**DOI:** 10.1016/j.ibneur.2023.08.003

**Published:** 2023-08-21

**Authors:** Radwa Khalil, Ahmed A. Karim, Ben Godde

**Affiliations:** a School of Business, Social and Decision Sciences, Constructor University, Bremen, Germany; b Department of Psychiatry and Psychotherapy, University Clinic Tübingen, Tübingen, Germany; c Department of Health Psychology and Neurorehabilitation, SRH Mobile University, Riedlingen, Germany

**Keywords:** Current Amplitude, Tactile, tDCS, Grating Orientation, Primary Somatosensory Cortex

## Abstract

**Background:**

Transcranial direct current stimulation (tDCS) is a frequently used brain stimulation method; however, studies on tactile perception using tDCS are inconsistent, which might be explained by the variations in endogenous and exogenous parameters that influence tDCS.

**Objectives:**

We aimed to investigate the effect of one of these endogenous parameters—the tDCS amplitude—on tactile perception.

**Methods:**

We conducted this experiment on 28 undergraduates/graduates aged 18–36 years. In separate sessions, participants received 20 min of 1 mA or 1.5 mA current tDCS in a counterbalanced order. Half of the participants received anodal tDCS of the left SI coupled with cathodal tDCS of the right SI, and this montage was reversed for the other half. Pre- and post-tDCS tactile discrimination performance was assessed using the Grating Orientation Task (GOT). In this task, plastic domes with gratings of different widths cut into their surfaces are placed on the fingertip, and participants have to rate the orientation of the gratings.

**Results:**

Linear modeling with amplitude, dome, and session as within factors and montage as between factors revealed the following: significant main effects of grating width, montage, and session and a marginally significant interaction effect of session and amplitude. Posthoc t-tests indicated that performance in GOT improved after 1 mA but not 1.5 mA tDCS independent of the montage pattern of the electrodes.

**Conclusion:**

Increasing the stimulation amplitude from 1 mA to 1.5 mA does not facilitate the tDCS effect on GOT performance. On the contrary, the effect seemed more robust for the lower-current amplitude.

## Highlights

• Dual-hemisphere somatosensory cortex (tDCS) improves tactile orientation discrimination (TOD).
• Increasing the stimulation amplitude from 1 mA to 1.5 mA does not facilitate the tDCS effect on TOD; however, 1 mA is more effective than 1.5 mA.
• The electrode montage did not influence the stimulation effect.

## Introduction

Transcranial direct current stimulation (tDCS) is a recently developed non-invasive brain stimulation technique that allows the induction of brain plasticity and subsequent modification of sensory, perceptual, motor, cognitive, and behavioral functions (Cole et al., 2018, Rumpf et al., 2018). The rationale beyond is that positively charged stimulation (anodal-tDCS (a-tDCS)) causes depolarization of superficial cortical neurons, while negatively charged stimulation (cathodal-tDCS (c-tDCS)) causes hyperpolarization (Nitsche et al., 2008, Nitsche et al., 2007, Nitsche et al., 2003). Among its numerous promising effects, tDCS has been shown to modulate sensory and tactile perception and discrimination; nevertheless, these effects reveal considerable heterogeneity (Parianen Lesemann et al., 2015). This variability of neurophysiological and behavioral tDCS effects can be attributed to extrinsic (methodological) factors, such as stimulation amplitude or electrode montage, and intrinsic (physiological) factors, such as individual differences in the susceptibility to tDCS (Chew et al., 2015, Nitsche et al., 2015, Polanía et al., 2018).

Several studies have revealed the effects of tDCS on performance in the tactile grating orientation discrimination task (GOT), but they are also rather heterogeneous. In the study by Ragert et al. (2008), a-tDCS stimulation with 1 mA amplitude over SI improved tactile spatial acuity in the contralateral hand compared to sham exposure. These effects were relatively short-lasting (about 40 min after the end of the stimulation period) and were not observed in the hand that was ipsilateral to SI. Hilgenstock et al. (2016) replicated these findings with 2 mA a-tDCS; there was an increase at the a-tDCS site (compared to the control group), as measured with functional magnetic resonance imaging (fMRI). Hirtz et al. (2018) further reported that repeated a-tDCS with 2 mA enhanced tactile spatial acuity of the contralateral index finger and found a transfer of the effect to the homologous ipsilateral index finger.

Contrary to these studies, Saito et al. (2019) and Godde et al. (2020) applied a-tDCS with 0.75 and 1 mA amplitudes, respectively, and did not find effects on GOT thresholds with the contralateral index finger. The study by Godde et al. (2020) only reported reduced tactile detection thresholds. Fujimoto et al., 2017, Fujimoto et al., 2014 reported reduced GOT thresholds in two independent studies using 1 or 2 mA stimulation amplitudes. Some studies systematically examined amplitude effects in the motor area, measuring motor-evoked potentials or brain activity after tDCS with different amplitudes. While an early study reported enhanced efficacy of tDCS with increasing stimulation intensity between 0.2 and 1 mA (Nitsche & Paulus, 2000), more recent studies revealed non-linear dose-response relationships. These studies were characterized by equal or even larger effects of low (0.5 or 1 mA) compared to higher (1.5 or 2 mA) intensities on motor cortical excitability (Jamil et al., 2017) or by stronger effects of lowest and highest (0.2 and 2 mA or 1 and 3 mA) compared to intermediate (0.5 and 2 or only 2 mA, respectively) intensities (Agboada et al., 2020, Chew et al., 2015, Mosayebi Samani et al., 2019). Concerning tDCS over the somatosensory cortex and its influences on tactile perception, to our knowledge, no study has directly compared the effects of different current amplitudes within the same study.

Regarding electrode montage, Fujimoto et al., 2017, Fujimoto et al., 2014 contrasted uni-hemispheric a-tDCS with dual-hemispheric tDCS. In their earlier study, these authors showed significantly better GOT performance after 1 mA dual-hemisphere tDCS than uni-hemisphere or sham tDCS conditions (Fujimoto et al., 2014). In the latter study, 2 mA dual-hemispheric tDCS was applied over the left (a-tDCS) and right (c-tDCS) parietal operculum (including the secondary somatosensory cortex). This study revealed that the bi-hemispheric stimulation was more effective than the uni-hemispheric stimulation, indicating that the ipsilateral parietal operculum inhibits tactile discrimination through inter-hemispheric inhibition (Fujimoto et al., 2017). A study by Yau et al. (2014) showed that GOT performance was improved with a-tDCS over the visual cortex but not the auditory cortex. Conversely, a-tDCS over the auditory cortex, but not the visual cortex, resulted in more significant improvements in sensitivity to vibration frequency. Shinde et al. (2021) indicated that bihemispheric montage had advantages over unihemispheric montage. Waters et al. (2017) hypothesized that bihemispheric tDCS (2 mA, 25 min) increases hemisphere cooperation and decreases transcallosal inhibition due to ipsilateral motor control.

Extending on the literature described above, we further investigated to what extent stimulation amplitudes and electrode montages could modulate the effects of tDCS on tactile discrimination performance as measured with the GOT. To achieve this aim, we directly compared the effects of two different stimulation amplitudes (1 and 1.5 mA) on GOT thresholds with two opposing dual-hemispheric montages, namely C3 + C4- and C4 + C3- .C3 + C4- refers to activating the C3 and simultaneously inhibiting its right-hemispheric counterpart C4, while C4 + C3- indicates activating the C4 and inhibiting its right-hemispheric counterpart C3.

Studies utilizing amplitudes of 0.75 mA (Saito et al., 2019) and 1 mA (Godde et al., 2020) did not report an effect of tDCS over the contralateral somatosensory cortex on GOT using the right index finger, suggesting that 1 mA is at the lower threshold for such effects. Therefore, we expected a more robust effect of a-tDCS with a higher amplitude (1 versus 1.5 mA) on GOT thresholds. Regarding the electrode placements, according to Fujimoto et al., 2017, Fujimoto et al., 2014, a-tDCS of the contralateral accompanied by c-tDCS of the ipsilateral somatosensory cortex results in stronger effects than contralateral a-tDCS alone due to additional inhibition of transcallosal inhibitory connections between the homologs areas. Following this suggestion, we hypothesized that C3 + C4-, i.e., a-tDCS of the left (contralateral) and c-tDCS of the right (ipsilateral) somatosensory cortex might induce reliable effects on GOT on the right index finger through activation of the cortical finger representation (Hilgenstock et al., 2016). In contrast, the opposite montage (C4 + C3-) should result in reduced contralateral somatosensory cortical activation and, thus, no change in GOT performance.

## Methods

### Experimental procedure

**Fig. 1:**
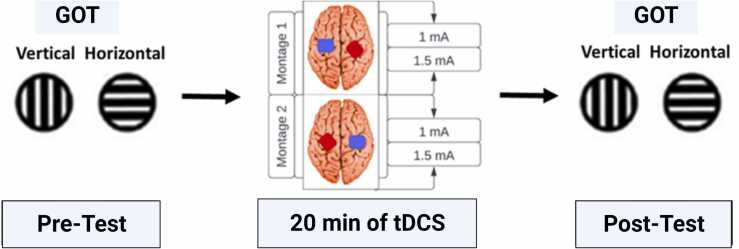
**Experimental setup.** In the grating orientation task (GOT), plastic domes with gratings of different widths were applied to the right index finger, and participants had to judge the orientation of the gratings. Performance in this task was measured before and after brain stimulation by tDCS. For transcranial direct current stimulation (tDCS), the active electrode (red, anode) was placed over the C3 position coupled with the cathodal electrode (blue, cathode) over C4, and vice versa.

Participants were assigned to two groups, receiving either a-tDCS over the left primary somatosensory cortex (SI) coupled with c-tDCS over the right SI or vice versa. Each participant attended two sessions, at intervals of one (minimum) to seven days (maximum), at similar times of the day. Each day, they received 20 min of tDCS with either 1 mA or 1.5 mA amplitudes in pseudo-randomized order. Participants completed the GOT pre- and post-tDCS (Fig. 1).

Participants relaxed in their chairs during the stimulation period without engaging in any task. The delay between stimulation and testing was 1–2 min, which covered the time needed to remove the electrodes and change the chair orientation. All the participants were verbally debriefed after the experimental session. Potential tDCS side effects were evaluated with a questionnaire immediately after the session.

### Participants

The study included 28 undergraduate and graduate students (aged 18–36 years) from Constructor University. All the participants underwent through eligibility screening for the tDCS procedure and gave written informed consent to participate in the experiment.The experimental protocol conformed to the Declaration of Helsinki and followed German law and ethical standards. None of the participants had previous experience with tDCS procedures. Handedness was formally tested with a modified Edinburgh Handedness Inventory (Oldfield, 1971). Participants with neurologic, medical, or psychiatric disorders were excluded. Participants were compensated for their contributions with either course credit or monetary compensation.

Participants were randomly divided into two stimulation groups: (1) cathodal stimulation (i.e., decreases in cortical excitability) of the left SI combined with anodal stimulation (i.e., increases in cortical excitability) of the right SI, or (2) anodal stimulation of the left SI combined with cathodal stimulation of the right SI. Each participant attended two sessions in which they received tDCS with either 1 mA or 1.5 mA amplitudes in randomized order.

### Transcranial direct current stimulation (tDCS)

A battery-driven stimulator (Schneider Electronic, Gleichen, Germany) applied tDCS over the right and left SI. The two electrodes were placed on the scalp using the 10–20 EEG system and secured with electrode positioning bands. An EEG cap was used to locate the C3 and C4 positions on the scalp, which were associated with the right and left SI, respectively (Grundmann et al., 2011, Ragert et al., 2008). A bilateral bipolar-balanced montage was applied, whereby we placed the electrodes symmetrically to activate the C3 and inhibit its right-hemispheric counterpart, C4, simultaneously or vice versa (Nasseri et al., 2015, Neuling et al., 2012). Such a bilateral configuration is particularly suitable for testing the balance in activation and inhibition between brain hemispheres (Khalil et al., 2020, Mayseless and Shamay-Tsoory, 2015). tDCS was applied through two saline-soaked sponge electrodes covering an area of 4 × 6 cm for 20 min with a 10 s ramp up and down (Nitsche et al., 2003, Nitsche and Paulus, 2000, Stagg and Nitsche, 2011).

We estimated the resulting electric field intensity using HD Explore software (Version 5.0.1; Soterix Medical, New York, NY, USA), applying a standard magnetic resonance imaging template (Borckardt et al., 2012, Klein et al., 2013). The current density in this study was 0.04 mA/cm^2^ for 1 mA, which is well above the threshold of 0.017 mA/cm^2^ under which one cannot modulate cortical excitability using tDCS according to Nitsche and Paulus (2000).

### Grating Orientation Test (GOT)

Tactile spatial discrimination was evaluated with the GOT by using eight different hemispherical plastic domes with equal widths of bars and grooves from 0.25 to 3 mm (indexed with letters A to H corresponding to widths 3, 2, 1.5, 1.25, 1, 0.75, 0.5, and 0.25 mm; JVP Domes, Stoelting, Wood Dale, IL, USA). In 10 blocks of 16 trials, participants should judge the orientation of the domes. Each dome was applied on the fingertip in a horizontal (perpendicular to the finger axis) and vertical (along the finger axis) orientation for 1–2 s in a randomized order. The percentage of correct responses was calculated per dome as a performance measure.

### Data Analysis

We performed all data analyses using Jamovi software version 2.4 (R Core Team, 2021, The jamovi project, 2022). The results were interpreted as statistically significant if the p-value was < 0.05 and as marginally significant with 0.5 < p < 1.0.

We performed a general linear model (GLM) fixed-effects analysis (Gallucci, 2019) after excluding two participants who performed at chance level (∼ 50 % correct responses) for the largest grating and did not reveal the typical pattern of decreasing performance with increasing task difficulty.^4^ Percentage correct was treated as the dependent variable, and SESSION (i.e., pre-and post-tDCS), DOME index representing the grating width (A–H), and current AMPLITUDE (1 or 1.5 mA) were treated as within-subject factors, while tDCS MONTAGE (C3 + C4- or C3-C4+) was treated as between-subject factor. Post hoc comparisons were performed for significant effects using Bonferroni correction. Data and analysis code for this study are available on request to the corresponding author.

## Results

The GLM analysis revealed significant main effects of DOME, MONTAGE, and SESSION (Table 1).

**Table 1:** Results of the statistical analysis using linear modeling.

ANOVA Omnibus tests					
	SS	df	F	p	η^2^p
**Model**	**21.6857**	**63**	**21.969**	< .001***	**0.625**
**Session**	**0.19451**	**1**	**12.414**	< .001***	**0.015**
**Dome**	**20.7602**	**7**	**189.28**	< .001***	**0.614**
Amplitude	0.01684	1	1.0751	0.300	0.001
**Montage**	**0.09621**	**1**	**6.1402**	0.013*	**0.007**
Session ✻ Dome	0.08859	7	0.8077	0.581	0.007
**Session ✻ Amplitude**	**0.04724**	**1**	**3.0150**	**0.083**^**+**^	**0.004**
Dome ✻ Amplitude	0.02912	7	0.2655	0.967	0.002
Session ✻ Montage	0.00748	1	0.4774	0.490	0.001
Dome ✻ Montage	0.06920	7	0.6310	0.731	0.005
Amplitude ✻ Montage	0.00457	1	0.2915	0.589	0.000
Session ✻ Dome ✻ Amplitude	0.06401	7	0.5836	0.770	0.005
Session ✻ Dome ✻ Montage	0.04205	7	0.3834	0.912	0.003
Session ✻ Amplitude ✻ Montage	1.64e-4	1	0.0105	0.919	0.000
Dome ✻ Amplitude ✻ Montage	0.06872	7	0.6265	0.734	0.005
Session ✻ Dome ✻ Amplitude ✻ Montage	0.01037	7	0.0946	0.999	0.001
Residuals	13.03597	832			
Total	34.72169	895			

*Note.*^*+*^ p < 0.1, * p < .05, ** p < .01, *** p < .001.

**Fig. 2:**
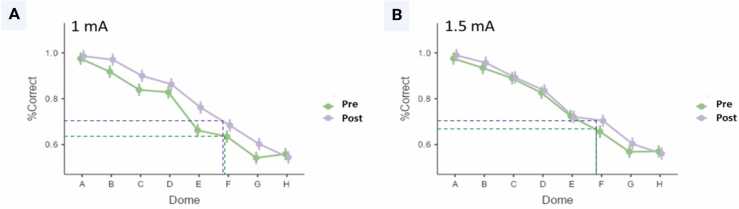
Effects of tDCS (pre-and post-tDCS) on GOT using two current amplitudes (1 mA and 1.5 mA, Panels A and B, respectively). Means and standard errors (SE) are shown.

The correct percentage probability decreased with decreasing spacing width but was higher after stimulation than before, indicating a reduction of the discrimination threshold. Consequently, the 75 % threshold was shifted to smaller dome widths. The effect was particularly strong for dome E, near the average threshold. Effects were stronger for 1 mA than 1.5 mA for the domes with larger distances, but the effects were similar at smaller distances (Fig. 2).

While montage groups differed in performance at baseline, the right anodal montage (C3-C4+) had lower baseline thresholds than the left anodal montage (C3 + C4-). However, MONTAGE did not modulate the tDCS effect, as indicated by a missing interaction effect of MONTAGE and SESSION. A marginally significant interaction of SESSION and AMPLITUDE indicated that stimulation effects at 1 mA might have been more robust than at 1.5 mA. Post hoc comparison revealed a significant difference between the two sessions (i.e., pre-and post-tDCS for 1 mA (t (832) = − 3.719, *p*_*bonf*_ = .001) but not for 1.5 mA (t (832) = − 1.264; *p*_*bonf*_ *= 1.0*)).

## Discussion

We aimed to investigate to what extent the effects of tDCS on GOT depend on stimulation amplitude (1 mA versus 1.5 mA) and montage (C3 + C4- versus C4 + C3-). We found that 1 mA improved tactile orientation perception and that this effect was more robust than 1.5 mA stimulation amplitude. However, no montage effect indicated that both right and left anodal stimulation facilitated tactile discrimination performance.

Ceiling effects are unlikely in light of the observation that higher amplitude did not result in stronger modulation of tactile performance for two reasons. First, performance measured as % correct responses was below 100 % for all domes for both amplitudes pre and post-tDCS. Second, the randomized crossover design would control for such ceiling effects as it should be specific for participants but not amplitudes.

Moreover, our results are in line with recent findings from the motor domain revealing non-linear does-response relationships where higher intensities do not consistently result in stronger effects (Agboada et al., 2020, Chew et al., 2015, Mosayebi Samani et al., 2019). Mosayebi-Samani et al. (2019) explain such non-linearity with Ca^2+^ channel dynamics in animal experiments to have non-linear modulatory effects on synaptic plasticity (Lisman, Misonou et al., 2004). Stronger fields may, therefore, cause network activity, which inhibits additional potentiation through homeostatic processes (Puri et al., 2016, Ranieri et al., 2012). Thus, the intended effects of tDCS may change from excitatory to inhibitory depending on stimulation time and/or intensity (Batsikadze et al., 2013, Monte-Silva et al., 2013). Higher amplitudes could cause more potential changes under the anode, activate intracortical inhibition from neighboring cortical columns, and counteract the higher neural activity in the core module representing the tested finger. However, a further increase in the amplitude would thus not be associated with a stronger effect. The bi-hemispheric montage might have contributed to the interpretation of our findings; higher activation in one hemisphere strengthens interhemispheric homotopic connections, balancing local excitation and inhibition.

Still, the underlying physiological mechanisms require further systematical exploration in future studies – particularly, in older adults who usually reveal less intracortical inhibition (Dinse et al., 1997, Dinse et al., 2006).

After Fujimoto et al. (2017), one would have expected that ipsilateral anodal stimulation would facilitate the effects of contralateral cathodal stimulation through interhemispheric connections and thus would not change the GOT thresholds. Nevertheless, the effects were similar for both montages in our study. Tazoe et al. (2014) reported that bilateral tDCS increased interhemispheric inhibition (IHI) from the facilitated (a-tDCS) side M1 to the inhibited (c-tDCS) side M1 and attenuated IHI in the opposite direction. According to a concurrent tDCS-fMRI study by Shinde et al. (2021), c-tDCS in a bihemispheric montage might operate as an excitatory stimulus rather than an inhibitory one. Sehm et al. (2013) confirmed reduced interhemispheric functional connectivity after bilateral motor cortex stimulation. One might speculate that reduced IHI from the ipsi- to the contralateral somatosensory cortex may have contributed to enhanced activation and, thus, better discrimination; however, such effects would hardly explain similar effects in both stimulation montages. In a study on creative cognition in bilateral montages, we reported similar effects of right and left anodal inferior frontal cortical stimulation on inhibitory control and divergent thinking (Khalil et al., 2020).

Consequently, more systematic research on the neural level is demanded to identify the intra- and interhemispheric neural connections and pathways responsible for inducing the effects of a-tDCS and c-tDCS in uni- and bilateral tDCS montages. In early animal studies using glass micropipettes to polarize the cortex electrically, Bindman et al. (1964) demonstrated that current orientation per se does not predict the after-effects of polarization. Instead, a distinction is necessary between the effects of external currents on various types of neurons and polarization-induced changes in specific neurons, which contribute to ongoing responses (Purpura & McMurtry, 1965). Therefore, there is a differential impact of tDCS on particular neuron types with implications for their functions (Giordano et al., 2017).

Ye et al. (2022) recently published a review on the neuronal parameters that influence how tDCS affects neuromodulation at multidimensional levels, including the tissue, cellular, and single ion channel levels. At a cellular (microscopic) level, the acute effects of externally applied direct currents also depend on neuron morphology, the (layer-specific) orientation of excitable neural elements in the induced electrical field (Rahman et al., 2013, Reato et al., 2013), stimulation intensity and duration, and the degree of spontaneous dendritic and somatic activity (Bikson et al., 2004, Fritsch et al., 2010, Radman et al., 2009, Rahman et al., 2013, Tranchina and Nicholson, 1986). At this level, glutamate is released by pyramidal neurons and thalamic afferents, whereas a variety of interneurons mainly release GABA (McCormick, 1992, Nicoll et al., 1990). Animal experiments using brain slices suggest that pyramidal neurons in the fifth layer are the most sensitive to the weak electric fields applied over the skull surface (Radman et al., 2009). Thus, a-tDCS and c-tDCS are expected to increase and decrease the membrane potential of pyramidal neurons, thereby altering the glutamatergic tone in the cortex. However, glutamate levels also depend on input from thalamic projections and interneurons. Consequently, tDCS modifies thalamocortical synapses through glutamate release from sensory afferents. As pyramidal neurons project to different types of interneurons, the modulation of glutamate levels is expected to correlate with GABA release. However, a recent computational modeling study based on in vivo experimental data proposed that tDCS may induce opposing effects on different types of interneurons (Molaee-Ardekani et al., 2013), resulting in a complex balance of excitation and inhibition. Yet, the effects observed in well-controlled in vitro or in vivo animal experiments with relatively uniform and homogeneous currents may be challenging to reproduce straightforwardly in human studies.

In conclusion, our findings highlight the impact of tDCS amplitudes for effects on grating orientation discrimination thresholds, with more robust effects for the lower amplitude, indicating the necessity to consider the current amplitude as a crucial tDCS factor for GOT beyond a simple linear dose-response function.

Noting the heterogeneity in the literature, more empirical research focusing on different current amplitudes is necessary to better understand the appropriate current amplitude that should be applied (López-Alonso et al., 2014). We propose more systematic empirical research on quantifying current amplitude effects on GOT performance in humans regardless of approach and outcome measure, which will benefit from including computational current flow models (Bestmann et al., 2015, Esmaeilpour et al., 2018).

### Limitations and future directions

This study has several limitations. First, it was a single-blind study, and the experimenter’s knowledge of the condition at each experiment may have introduced bias in testing (Gandiga et al., 2006). Second, the experimenter tested the participants manually, using a semi-automatic device, as exists for GOT (Ragert et al., 2008), which might have compensated for the lack of double blinding in this study. Third, the effect sizes were relatively small.

Although the physiological basis and mechanisms of tDCS are emphasized in many studies using animal and human models, these studies were relatively segregated (Morya et al., 2019). Several questions remain open: How can models of current flows in the human brain using tDCS be combined with animal models of the physiological impact of these current amplitudes? To what extent is this approach subject to assumptions about the spatial extent of current flow? How can the assumptions implicit in conventional dose-testing studies be made more explicit? In dose-response studies, can computational models be used to retrospectively predict brain current intensity across individuals for a fixed applied current? Has the research scale on tDCS efficacy outstripped the understanding of dose response? What is the precise effect of tDCS on neural information transmission (Bikson et al., 2004, Bikson et al., 2015; Bikson & Dmochowski, 2020)?

For instance, the current flow direction differently affects dendrites (Aspart et al., 2018, Kronberg et al., 2017), soma (Chan et al., 1988, Radman et al., 2009), axon terminals (Bikson et al., 2004, Chakraborty et al., 2018, Kabakov et al., 2012, Rahman et al., 2013), glia (Monai et al., 2016, Monai and Hirase, 2018), and endothelial cells (Cancel et al., 2018). Anodal stimulation of dendrites polarizes the apical dendritic layer and depolarizes the pyramidal cortical neurons' soma; the reported tDCS outcomes are related to modified excitability (Bikson et al., 2004, Chan and Nicholson, 1986, Huang et al., 2017, Purpura and McMurtry, 1965), neuroplasticity (Gartside, 1968, Kronberg et al., 2017) and neural network oscillation (D’Andola et al., 2018, Reato et al., 2010). Therefore, providing an integrative approach combining animal and human studies will allow validation of how current flow models can explain the variability in tDCS outcomes.

## Ethics approval and consent to participate

We declare that all experiments on human participants followed the Declaration of Helsinki and that all procedures were executed with the participants' adequate understanding and written consent. We certify that formal approval to conduct the experiments described has been obtained from Constructor University's human subjects review board and could be provided upon request.

## Funding

This research was supported by 10.13039/501100001659Deutsche Forschungsgemeinschaft (DFG: GO 802/12-1 and KA 4267/3).

## CRediT authorship contribution statement

**Radwa Khalil:** Conceptualization, Methodology, Visualization, Data curation, Formal analysis, Writing – original draft, Writing – review & editing. **Ahmed A. Karim:** Conceptualization, Funding acquisition, Methodology. **Ben Godde:** Conceptualization, Funding acquisition, Methodology, Visualization, Writing – review & editing.

## Conflicts of Interest

No conflicts of interest, financial or otherwise, are declared by the author(s).

## Footnotes

4 We also performed a mixed-effect analysis because groups had different baseline GOT levels. However, this analysis revealed only a marginal random effect and that a fixed-effect analysis would be more appropriate.
